# Comparative Effects of Chloride Channel Inhibitors on LRRC8/VRAC-Mediated Chloride Conductance

**DOI:** 10.3389/fphar.2017.00328

**Published:** 2017-05-31

**Authors:** Jonas Friard, Michel Tauc, Marc Cougnon, Vincent Compan, Christophe Duranton, Isabelle Rubera

**Affiliations:** ^1^LP2M CNRS-UMR7370, LabEx ICST, Medical Faculty, Université Côte d’AzurNice, France; ^2^Institut de Génomique Fonctionnelle, Centre National de la Recherche Scientifique, Institut National de la Santé et de la Recherche Médicale, Université de MontpellierMontpellier, France

**Keywords:** DCPIB, CFTR_inh_-172, GlyH-101, PPQ-102, NFA, T16A_inh_-A01

## Abstract

Chloride channels play an essential role in a variety of physiological functions and in human diseases. Historically, the field of chloride channels has long been neglected owing to the lack of powerful selective pharmacological agents that are needed to overcome the technical challenge of characterizing the molecular identities of these channels. Recently, members of the LRRC8 family have been shown to be essential for generating the volume-regulated anion channel (VRAC) current, a chloride conductance that governs the regulatory volume decrease (RVD) process. The inhibitory effects of six commonly used chloride channel inhibitors on VRAC/LRRC8-mediated chloride transport were tested in wild-type HEK-293 cells expressing LRRC8 proteins and devoid of other types of chloride channels (CFTR and ANO1/2). We explored the effectiveness of the inhibitors using the patch-clamp whole-cell approach and fluorescence-based quantification of cellular volume changes during hypotonic challenge. Both DCPIB and NFA inhibited VRAC current in a whole-cell configuration, with IC_50_ values of 5 ± 1 μM and 55 ± 2 μM, respectively. Surprisingly, GlyH-101 and PPQ-102, two CFTR inhibitors, also inhibited VRAC conductance at concentrations in the range of their current use, with IC_50_ values of 10 ± 1 μM and 20 ± 1 μM, respectively. T16A_inh_-A01, a so-called specific inhibitor of calcium-activated Cl^-^ conductance, blocked the chloride current triggered by hypo-osmotic challenge, with an IC_50_ of 6 ± 1 μM. Moreover, RVD following hypotonic challenge was dramatically reduced by these inhibitors. CFTR_inh_-172 was the only inhibitor that had almost no effect on VRAC/LRRC8-mediated chloride conductance. All inhibitors tested except CFTR_inh_-172 inhibited VRAC/LRRC8-mediated chloride conductance and cellular volume changes during hypotonic challenge. These results shed light on the apparent lack of chloride channel inhibitors specificity and raise the question of how these inhibitors actually block chloride conductances.

## Introduction

For a long time, anion permeabilities have been regarded as playing a minor role in membrane conductance and have been generally believed to simply follow the electrochemical gradient imposed by the potassium-mediated membrane potential. This situation has been aggravated by the lack of powerful and selective inhibitors, which has led to difficulty in characterizing the molecular identities of these Cl^-^ channels. However, the situation changed drastically in the late 1980s when mutations of the chloride channel CFTR (Riordan et al., 1989) were linked to cystic fibrosis, one of the most frequently inherited human diseases. In addition to CFTR, another family of chloride channels, ClC, has been identified in mammals by homology screening of the chloride channel ClC-0 from the torpedo fish (Jentsch et al., 1990). A mutation in ClC-1 (the principal skeletal muscle Cl^-^ channel) was reported to generate myotonia (Koch et al., 1992).

Unfortunately, despite numerous efforts to characterize the proteins that constitute the CaCC and the VRAC, their molecular identities have long remained unknown. In 2008, three independent laboratories (Caputo et al., 2008; Schroeder et al., 2008; Yang et al., 2008) finally identified anoctamin-1 and -2 (ANO1 and ANO2, also called TMEM16A and TMEM16B, respectively), two members of the large TMEM16 family, as good candidates for CaCC-mediated conductance. Electrophysiological approaches using various heterologous transfected cell models demonstrated that ANO1/2 exhibit strong biophysical similarity to the well-described CaCC.

Despite its ubiquitous expression, an even longer time was required to precisely identify the molecular identity of the VRAC channel (also termed volume-sensitive outwardly rectifying, VSOR Cl^-^ channels or volume-sensitive organic osmolyte/anion channel, VSOAC). This Cl^-^ conductance has been recorded in virtually all cell types in which cellular swelling occurs in response to a decrease in the external osmolality (Grinstein et al., 1982; Worrell et al., 1989; Hunter, 1990); additionally, for review, see Lang et al. (1998) and Pedersen et al. (2016). The biophysical signature of the VRAC current is characterized by (i) activation upon exposure to hypotonic extracellular solution (ii) significant inactivation of the current at positive potentials upon stimulation and (iii) an Eisenman selectivity type 1 profile: SCN^-^>I^-^>Cl^-^>${\mathrm{NO}}_{3}^{–}$>Br^-^>lactate>glutamate; for review, see Pedersen et al. (2016). The molecular identity of the VRAC remained elusive until 2014, when two independent groups (Qiu et al., 2014; Voss et al., 2014), using genome-wide screening coupled with iodide-sensitive fluorescent probe detection, identified LRRC8A (leucine-rich repeat containing 8 A) as a necessary component of the VRAC-mediated conductance. VRACs are heteromers of LRRC8A and at least one other LRRC8 isoform (LRRC8B – LRRC8E) (Voss et al., 2014). This result suggests that there exists a large variety of differently composed VRACs that may differ substantially in their ability to conduct organic compounds such as osmolytes and neurotransmitters (Planells-cases et al., 2015; Lutter et al., 2017). Thus, the identification of inhibitors that are specific for certain subunit combinations will be very useful (Planells-cases et al., 2015).

In addition to its unambiguous involvement in the process of RVD, VRAC conductance can be activated under iso-osmotic conditions (i.e., in the absence of cell swelling) both (i) during exposure of cells to reduced intracellular ionic strength (Nilius et al., 1998; Syeda et al., 2016) (ii) during purinergic pathway stimulation (Barriere et al., 2003; Belfodil et al., 2003) and (iii) during apoptosis volume decrease AVD (Maeno et al., 2000; Wang et al., 2005; L’hoste et al., 2009). For decades, inhibitors have been used as tools to investigate the roles and functions of chloride channels, especially in the case of the Cl^-^ channel CFTR. However, the first generation of inhibitory molecules, including DPC (diphenylamine-2-carboxylic acid), NPPB (5-nitro-2-(3-phenylpropylamino)benzoic acid), glibenclamide, DIDS (4,4′-diisothiocyanatostilbene-2,2′-disulfonic acid), and SITS (4-acetamido-4′-isothiocyanato-2,2′-stilbenedisulfonic acid), exhibited no ability to selectively inhibit a single family of chloride channels. This was particularly true for NPPB, which non-selectively inhibits CFTR, ClC-x, CaCC- and VRAC-mediated chloride conductance. For more than 10 years (1990–2000), the characterization of an “unknown” Cl^-^ conductance was based on its differential sensitivity to DIDS versus other Cl^-^ channel inhibitors; CFTR-mediated conductance was considered not to be sensitive to DIDS compared to other types of chloride channel inhibitors.

In the early 2000s, two new and potent inhibitors of CFTR, CFTR_inh_-172 and GlyH-101, were described. These compounds are now widely used to specifically inhibit CFTR (Ma et al., 2002; Muanprasat et al., 2004). However, despite some evidence, the effects of both drugs on other types of Cl^-^ conductance proteins have been partially overlooked (Caputo et al., 2008; Melis et al., 2014). More recently, in an effort to identify new inhibitors of CFTR, the compound PPQ-102 was demonstrated to inhibit CFTR conductance and to prevent expansion of cysts in a kidney organ culture model of polycystic kidney disease (Tradtrantip et al., 2009).

DCPIB (4-(2-butyl-6,7-dichlor-2-cyclopentyl-indan-1-on-5-yl) oxybutyric acid), a derivative of etacrynic acid, was shown to be a good inhibitor of the VRAC current at concentrations that putatively do not inhibit CFTR, ClC or CaCC (Decher et al., 2001) and is currently one of the most potent known inhibitors of VRAC-mediated conductance. Another compound, niflumic acid (NFA), has been described as one of the most potent inhibitors targeting CaCC conductance (White and Aylwin, 1990). However, the wide range of reported NFA IC_50_ values [varying from micromolar (Romanenko et al., 2010) to hundreds of micromolar (Ledoux et al., 2005)] raises some concerns about its specificity. Unfortunately, NFA has also been shown to block VRAC-mediated conductance in various cellular models (Worrell et al., 1989; Kelly et al., 1994). In 2008, the identification of TMEM16A/ANO1 as a candidate of the CaCC was rapidly followed by the development of a putative selective inhibitor of this chloride current, T16A_inh_-A01 (De La Fuente et al., 2008). Currently, this inhibitor, as well as some others (CaCC_inh_-A01 and MONNA), are being used to explore the contribution of CaCC/ANO1 in physiological models (Namkung et al., 2013; Sauter et al., 2015). However, a recent study (Boedtkjer et al., 2015) questioned the specificity of these inhibitors for CaCC/ANO1 chloride current, because the authors observed similar inhibitory effects under experimental conditions in which the Cl^-^ gradient was blunted.

In this study, we explored the efficacy of six commonly used chloride channel inhibitors on VRAC/LRRC8 conductance using the patch-clamp technique and characterized their actions on the VRAC-related volume regulatory function. The effect of each inhibitor was tested using two experimental approaches, combining patch-clamp whole cell recordings and indirect fluorescence measurements of volume change. We chose a panel of inhibitors that interact with three of the four main chloride channel families that have been described in mammalian epithelial cells: CFTR_inh_-172, GlyH-101 and PPQ-102, which are classically used to inhibit CFTR-mediated Cl^-^ current; T16A_inh_-A01 and NFA, which target CaCC/ANO1 chloride-mediated transport; and, finally, DCPIB, which is the most potent and selective known inhibitor of the VRAC/LRRC8-mediated Cl^-^ conductance.

## Materials and Methods

### Inhibitors

CFTR_inh_-172, DCPIB and T16A_inh_-A01 (Sigma–Aldrich) were prepared as 20 mM stock solutions in DMSO and stored at -20°C. GlyH-101 (Tocris), PPQ-102 (Calbiochem) and NFA (Sigma–Aldrich) were prepared as 10 mM stock solutions in DMSO and stored at -20°C.

### Cell Culture

We used the immortalized cell line HEK-293 (wild-type) and, for negative control experiments, HEK-293 LRRC8A KO [kindly provided by Prof. TJ Jentsch, Berlin, Germany (Voss et al., 2014)]. The cells were classically cultured in DMEM culture medium containing 10% serum and penicillin (100 U/ml), streptomycin (100 μg/ml). The cultures were maintained in a water-saturated atmosphere of 5% CO_2_/95% air at 37°C.

### qRT-PCR Analysis

Reverse transcription was performed using 2 μg of RNA samples, M-MLV-RT (Promega) and random primers (250 ng/μl, Roche Diagnostics). The primer sequences were designed using Primer Express software (Applied Biosystems, Courtaboeuf, France) and tested for their specificity, efficiency, reproducibility and dynamic range. For quantitative PCR, the final reaction volume was 10 μl; SYBR green master mix (Eurogentec, Angers, France) and 100 nM of each primer were used. The assays were run on a StepOnePlus Real-Time PCR System (Applied Biosystems). The expression levels of selected human genes were quantified by the comparative -ΔCt method using 36B4 as the reference gene. The primer sequences used were:

LRRC8A: s- GGGTTGAACCATGATTCCGGTGAC; as- GAAGACGGCAATCATCAGCATGAC
LRRC8B: s- ACCTGGATGGCCCACAGGTAATAG; as- ATGCTGGTCAACTGGAACCTCTGC
LRRC8C: s- ACAAGCCATGAGCAGCGAC; as- GGAATCATGTTTCTCCGGGC
LRRC8D: s- ATGGAGGAGTGAAGTCTCCTGTCG; as- CTTCCGCAAGGGTAAACATTCCTG
LRRC8E: s- ACCGTGGCCATGCTCATGATTG; as- ATCTTGTCCTGTGTCACCTGGAG
CFTR: s- GCAGCCTTACTTTGAAACTC; as- AACAGCAATGAAGAAGATGAC
TMEM16A: s- GGCATATTCCAGAGGAGTCAA; as- TCCATGTCAGCTTCACTTTGTC
TMEM16B: s- GCCAGGGATCCATCTTTGT; as- CCTGCTTTGATCTCGTACATTTT
TMEM16C: s- GCAGAGAGGCTGAATATCAGGA; as- GCATCCTGCCCATTGATT
TMEM16D: s- TGACTGGGATTTGATAGACTGG; as- GCTTCAAACTGGGGTCGTAT
TMEM16E: s- TGGAAACATTAAAGAAGCCATTT; as- GAGTTTGTCCGAGCTTTTCG
TMEM16F: s- AGGAATGTTTTGCTACAAATGGA; as- GTCCAAGGTTTTCCAACACG
TMEM16G: s- GCTCTGTGGTGATCGTGGT; as- GGCACGGTACAGGATGATAGA
TMEM16H: s- GGAGGACCAGCCAATCATC; as- TGCTCGTGGACAGGGAAC
TMEM16J: s- CGGAAGTCAGGTAGGAGCAC; as- ATCCGGAGGCTCTCTTCG
TMEM16K: s- TTGTATCCAGGAAAATCATTGTTG; as- AAGCTTCTTCAGGGCTTCACT

### Patch-Clamp Measurements

The ruptured-whole-cell configuration of the patch-clamp technique was used to measure the activation of VRAC conductance during hypotonic experimental conditions. Cell currents and cell capacitances were recorded using an EPC 10 amplifier [HEKA Elektronik, Lambrecht (Pfalz), Germany]. The membrane potential of the cells was held at -50 mV, and 400-ms pulses from -100 to +100 mV were applied in 20-mV increments. The I/V relationships were expressed as the mean current amplitudes measured at all potentials at 6–10 ms after the pulse onset. The offset potentials between both electrodes were zeroed before sealing and corrected for liquid junction potentials as previously described (Duranton et al., 2002).

The pipette solution contained (in mM) 140 NMDGCl, 10 HEPES (pH 7.4, HCl), 5 EGTA and 5 MgATP (290 mOsm.l^-1^). The normal NMDGCl bath solution contained (in mM) 140 NMDGCl, 10 HEPES (pH 7.4, HCl), 1 CaCl_2_, 1 MgCl_2_, and ∼70 mannitol (340 mOsm.l^-1^). This solution was designed to prevent the spontaneous activation of VRAC currents. Hypo-osmotic NMDGCl solution (270 mOsm.l^-1^) was obtained by removing the mannitol from the NMDGCl bath solution.

Patch-clamp whole-cell recordings were performed in iso-osmotic bath solution (control condition), and the solution was rapidly exchanged to the hypo-osmotic solution. The hypotonic condition was maintained until the LRRC8/VRAC current had stabilized at its maximal value (3–6 min). The percent inhibition at each concentration of inhibitor was calculated at -100 mV holding potentials after at least 2 min of constant perfusion.

### Measurements of Cell Volume Change

Measurements of changes in cell volume were performed using the calcein fluorescence self-quenching method. Briefly, calcein at high concentrations undergoes spontaneous self-quenching, and its fluorescence intensity decreases with increasing calcein concentration (Capó-Aponte et al., 2005). The variation in the fluorescence due to self-quenching indirectly reflects the volume of the cell.

HEK-293 WT and HEK-293 LRRC8-KO cells were cultured on polylysine-coated 24-well plates for 2 days. The cells were then loaded for 40 min with a solution containing 10 μM calcein-AM (Sigma–Aldrich) and washed 3 times with 1 ml of isotonic medium containing (in mM) 145 NaCl, 5 KCl, 1 MgCl_2_, 1 CaCl_2_, 10 glucose, and 10 HEPES pH 7.4 (300 mOsm.l^-1^). Under this control experimental condition, basal fluorescence was measured every 90 s for 10 min using a plate reader (Ex: 460 nm, Em: 520 nm; Synergy HT, Biotek). After the baseline recording period (10 min), the medium was exchanged and replaced with iso- or hypo-osmotic bath solution adjusted to 200, 150, or 100 mOsm.l^-1^. The change in fluorescence induced by replacement of the medium was then recorded over the following 50 min. The experiments were performed under control conditions and in the presence of various concentrations of inhibitors. The percent inhibition resulting from exposure of the cells to solutions containing a concentration of 10 μM for each inhibitor was calculated after maintaining the cells in hypotonic bath solution in the presence of the inhibitor for 30 min.

### Data Analysis

Analysis of the inhibition curves was performed using GraphPad Prism software [Prism 6 version 6.01 (2012), ©2017 GraphPad Software, Inc, URL^1^]. Concentration-response curves, IC_50_ and Hill slope were fitted and determined using the following equations:

$$\begin{array}{c} \mathrm{Span}=\mathrm{Top}-\mathrm{Bottom}\\ \;y=\mathrm{Bottom}+\frac{\left(\mathrm{Top}-\mathrm{Bottom}\right)}{1+10^{{\left(LogIC50-x\right)}^{*}\mathrm{Hill}\;\mathrm{Slope}}} \end{array}$$

Statistical analysis was performed using R software [R version 3.2.3 (2015), ©2015, The R Foundation for Statistical Computing, URL^2^]. *P*-values less than 0.05 were considered significant (^∗^).

## Results

**FIGURE 1:**
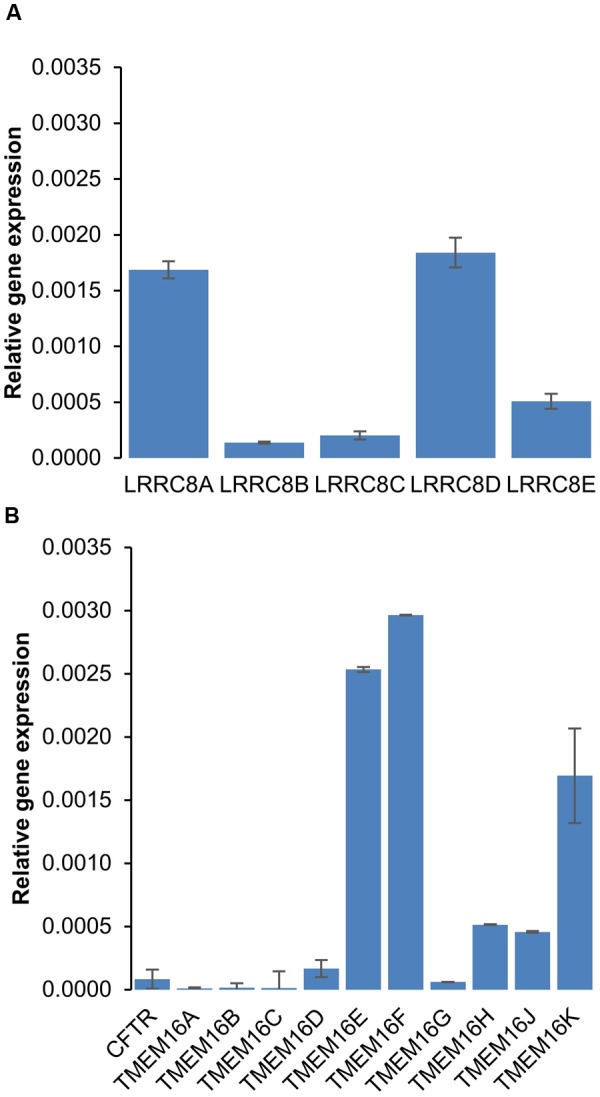
mRNA expression of chloride channels in HEK-293 cells. **(A,B)** Quantitative RT-PCR was used to measure the mRNA expression levels of LRRC8 subunits **(A)** and of the CFTR and TMEM16 families **(B)** compared to 36B4. The experiments were performed using 3 different cell cultures obtained from 3 different passages. The reported values are the mean ±SEM of 9 independent quantitative RT-PCR experiments.

To explore the effects of six different chloride channel inhibitors on LRRC8-mediated Cl^-^ transport, we used the HEK-293 wild-type cell line, which expresses LRRC8 proteins (**Figure 1A**) and lacks other known types of Cl^-^ channels. Quantitative PCR experiments confirmed that neither CFTR transcripts nor TMEM16A (ANO1) or TMEM16B (ANO2) transcripts were significantly expressed in this cell line (**Figure 1B**). By contrast, TMEM16E and F were significantly expressed. To functionally confirm that this cell line exhibited only VRAC/LRRC8 conductance, we recorded the variation in the chloride current in the whole cell configuration using symmetrical NMGCl pipette and bath solutions in the presence of high levels of cAMP (10 μM forskolin + 100 μM IBMX) or high intracellular free calcium (ionomycin, 1 μM, Supplementary Figure S1). Both of these experimental conditions failed to stimulate any chloride currents activated by phosphorylation (CFTR conductance) or by increased levels of intracellular free calcium (CaCC/ANO1-2 conductance).

To assess the sensitivity of the LRRC8A/VRAC current (I_Cl/swell_) to different inhibitors, we used two different and complementary experimental approaches. One approach was based on patch-clamp recording of the LRRC8/VRAC current (whole-cell configuration), and the other was based on the modulation of the cellular volume induced by activation of the RVD process.

**FIGURE 2:**
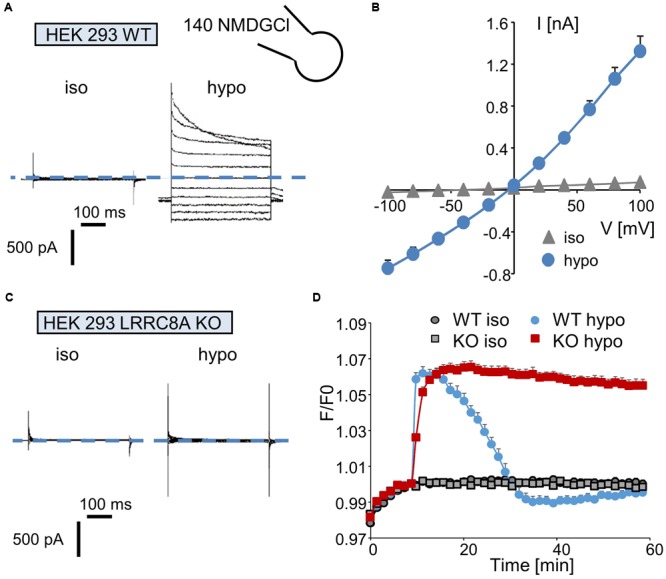
Quantification of I_Cl,swell_ and of the regulatory volume decrease (RVD) process in wild-type and LRRC8A KO HEK-293 cells. **(A,C)** Whole-cell currents recorded in WT **(A)** and LRRC8A KO **(C)** HEK-293 cells maintained under control conditions (in an iso-osmotic bath solution) or exposed to hypotonic medium for 5 min. The membrane potential was held at –50 mV, and currents were elicited by a train of 11 voltage steps (400 ms duration) between –100 and +100 mV in +20-mV increments. **(B)** Mean current/voltage relationships measured in HEK-293 WT recorded under iso-osmotic conditions (iso) and 5 min after exposure to hypotonic medium (hypo). Current values were measured 10 ms after the onset pulse. The reported values are the mean (±SEM) values obtained from 35 individual cells. **(D)** Quantification of RVD measured in WT and LRRC8A KO HEK-293 cells following a hypotonic challenge. The measurements were performed using calcein relative fluorescence quenching induced by changes in cellular volume. After a 10-min period for fluorescence stabilization, the bath solution was replaced by an iso-osmotic solution (300 mOsm.l^-1^) or by a hypo-osmotic solution (100 mOsm.l^-1^). Fluorescence emitted at 520 nm was measured every 90 s over a 60-min period.

We first recorded the activation of the I_Cl/swell_ conductance in the whole-cell configuration by exposing wild-type HEK-293 cells to a hypotonic solution. Replacing the isotonic NMDGCl bath solution (∼340 mOsm.l^-1^) with a hypotonic NMDGCl solution (270 mOsm.l^-1^) induced the activation of a Cl^-^ current that reached a maximal level within 3–5 min; this current displayed a reversal potential of 0 mV, corresponding to the symmetrical concentration of chloride ions (**Figure 2A**). The mean I/V curve recorded in 35 independent cells is shown in **Figure 2C**. Under this hypotonic challenge, the conductance was 7.69 ± 0.07 nS (-100 to -40 mV) and 12.91 ± 0.20 nS (+40 to +100 mV). Application of the same experimental protocol to HEK-293 LRRC8A KO cells failed to activate any chloride current (**Figure 2B**).

Next, we evaluated the involvement of LRRC8/VRAC Cl^-^ channels in the RVD process following a rapid decrease in the osmolality of the bath solution. Under control conditions (no change in osmolality, **Figure 2D**), the fluorescence of the calcein-AM probe was maintained at a constant level during the entire experiment in both wild-type and LRRC8A KO HEK-293 cells. By contrast, replacing the isotonic bath solution (300 mOsm.l^-1^) with a hypotonic solution (100 mOsm.l^-1^) induced a rapid increase in fluorescence (the maximal fluorescence occurred within 5 min, corresponding to the maximal cell swelling) followed by a decreasing phase reflecting the RVD process. As expected (**Figure 2D**) and as already demonstrated (Voss et al., 2014), HEK-293 LRRC8A KO cells failed to regulate their volumes and maintained high fluorescence values during the entire experiment.

Interestingly, in wild-type HEK-293 cells, replacing the isotonic bath solution with a 200 mOsm.l^-1^ solution failed to induce the RVD process (Supplementary Figure S2A), whereas replacing it with a 150 mOsm.l^-1^ solution resulted in a moderate and long-lasting period of regulation. Surprisingly, the absence of a polylysine coating on the plastic culture support markedly attenuated the cellular RVD process recorded under hypo-osmotic conditions in wild-type HEK-293 cells (100 mOsm.l^-1^, Supplementary Figure S2B).

Using these two experimental approaches, we next quantified the sensitivity of the LRRC8/VRAC Cl^-^ permeability to six different Cl^-^ channel inhibitors.

### DCPIB

**FIGURE 3:**
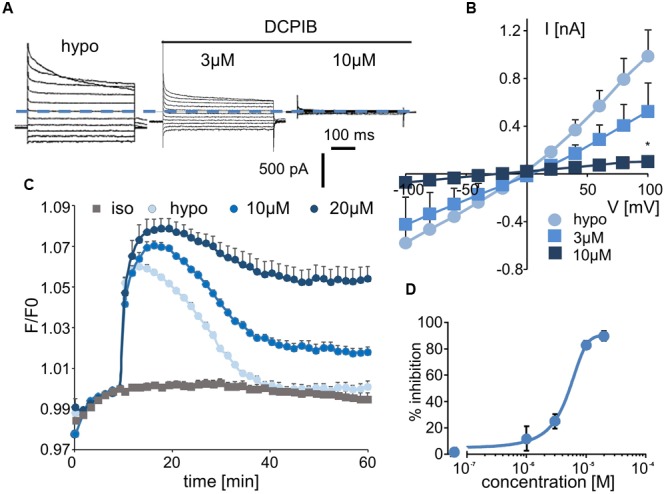
Effect of DCPIB on I_Cl,swell_ and on the RVD process. **(A)** Whole-cell current traces recorded from WT HEK-293 cells in hypotonic solution (hypo, 5 min) and in the presence of DCPIB at 3 and 10 μM. **(B)** Mean current/voltage relationships measured at 10 ms after the onset pulse corresponding to experiments performed as in **(A)** in the absence or presence of increasing concentrations of DCPIB (3 and 10 μM, *n* = 5 individual records for each concentration). **(C)** Inhibition by DCPIB (10 μM, *n* = 12; 20 μM, *n* = 8) of RVD following 100 mOsm.l^-1^ hypotonic challenge based on calcein relative fluorescence. **(D)** Dose-response inhibition curves calculated from whole-cell current traces **(A)** obtained from cells exposed to 1, 3, 10, and 20 μM DCPIB. The reported values are the mean ±SEM of 5 individual records obtained at each experimental concentration. The curves were calculated at –100 mV and permitted IC_50_ calculation.

Whole-cell experiments confirmed that DCPIB inhibits the volume-sensitive Cl^-^ current mediated by LRRC8/VRAC activation in a dose-dependent manner (**Figure 3A**). The corresponding I–V curves for the control condition and for three distinct concentrations of DCPIB are illustrated in **Figure 3B**. No voltage-dependent inhibition by DCPIB was observed. The calculated IC_50_ was 4.8 ± 1.2 μM (at -100 mV, *n* = 5), and more than 90% inhibition was observed at a DCPIB concentration of 20 μM (**Figure 3D**). Using the fluorescence approach, we also observed that inhibition of the RVD process by DCPIB was dose-dependent. Significant inhibition by DCPIB was measured at 10 μM (36 ± 3%, *n* = 8), whereas a concentration of 20 μM had the maximal effect (**Figure 3C**).

### CFTR_inh_-172, GlyH-101, and PPQ-102

**FIGURE 4:**
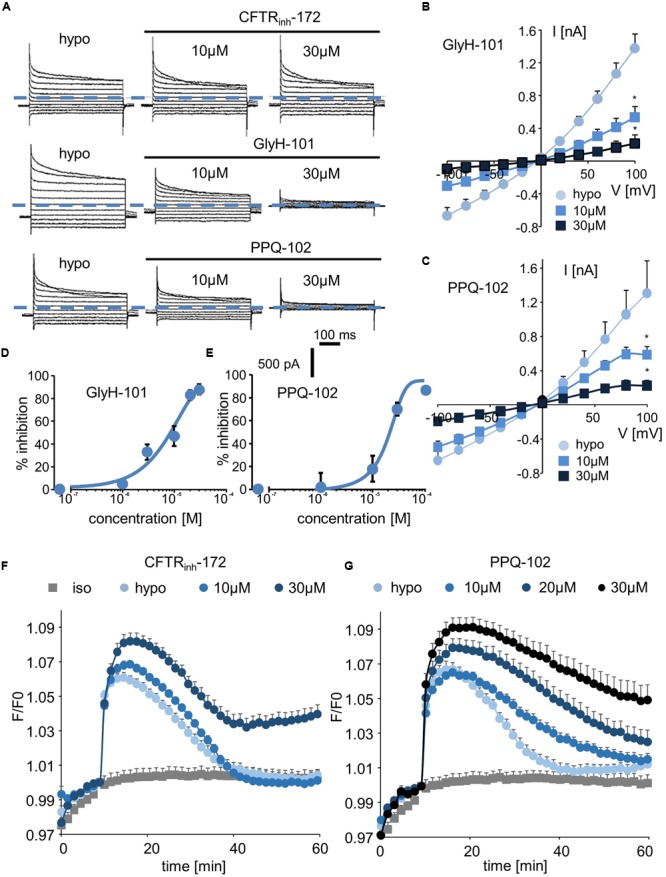
Effect of CFTR inhibitors (CFTR_inh_-172, GlyH-101, and PPQ-102) on I_Cl,swell_ and on the RVD process. **(A–C)** Whole-cell I_Cl,swell_ inhibition induced by CFTR_inh_-172 at 10 and 30 μM, GlyH-101 at 10 and 30 μM, PPQ-102 at 10 and 30 μM **(A)** and relative IV curves **(B,C)**. **(D,E)** Inhibition concentration curves permitting IC_50_ calculation for PPQ-102 and GlyH-101. The reported values are the mean ±SEM of 5 individual records obtained for each experimental concentration and each inhibitor. **(F,G)** Inhibition by CFTR_inh_-172 (10 μM, *n* = 10; 20 μM, *n* = 10) and PPQ-102 (10 μM, *n* = 12; 30 μM, *n* = 8) of RVD following 100 mOsm.l^-1^ hypotonic challenge based on calcein relative fluorescence.

We next explored the sensitivity of VRAC/LRRC8 chloride conductance to three inhibitors that are known to target CFTR-mediated chloride conductance. In whole-cell recordings, CFTR_inh_-172 exhibited almost no effect on hypotonic-activated Cl^-^ currents at concentrations of up to 10 μM; it had a minor effect at 20 μM (current traces, **Figure 4A**; I/V curve and dose/inhibition curve, Supplementary Figures S3A,B). Very modest inhibition was observed only at +80 and +100 mV at the end of the onset pulse.

By contrast, GlyH-101 and PPQ-102 inhibited VRAC/LRRC8 conductance in a dose-dependent manner with more than 85% inhibition at 20–30 μM, respectively (**Figures 4A–C**). The calculated IC_50_ values for GlyH-101 and PPQ-102 were 9.5 ± 1.1 μM (at -100 mV, *n* = 5, **Figure 4D**) and 19.6 ± 1.5 μM (at -100 mV, *n* = 5, **Figure 4E**), respectively.

Using the calcein-mediated fluorescence approach, we observed that inhibition of the RVD process by CFTR_inh_-172 occurred only at 30 μM and that lower doses were ineffective (**Figure 4F**), consistent with the results of the whole-cell experiments. For GlyH-101, the spontaneous fluorescence of this molecule at the wavelength used for calcein excitation precludes quantification of its inhibitory activity; under iso-osmotic conditions, addition of 10 μM GlyH-101 induced a large increase in fluorescence independently of any volume change (Supplementary Figure S4). By contrast, PPQ-102 inhibited the RVD process in a dose-dependent manner; a concentration of 10 μM was sufficient to produce significant inhibition (30 ± 6%, *n* = 12), whereas a concentration of 30 μM fully inhibited RVD (**Figure 4G**).

### T16A_inh_-A01 and Niflumic Acid (NFA)

**FIGURE 5:**
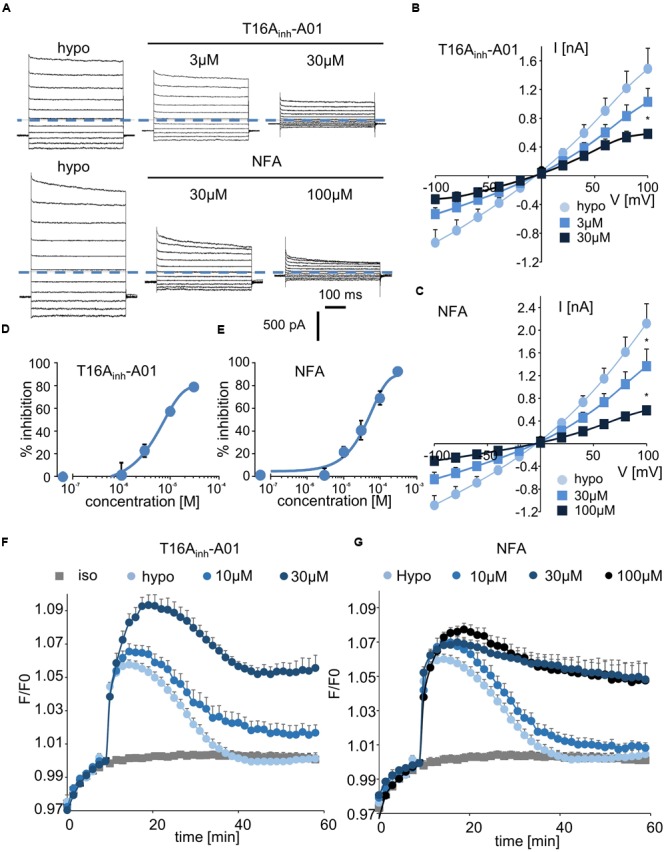
Inhibition of I_Cl,swell_ and RVD by CaCC inhibitors. **(A,C)** Whole-cell I_Cl,swell_ inhibition by T16A_inh_-A01 at 3 and 30 μM and by NFA at 30 and 100 μM **(A)** and the relative IV curves **(B,C)**. **(D,E)** Inhibition concentration curves permitting IC_50_ calculation for T16A_inh_-A01 and NFA. The reported values are the mean ±SEM of 5 individual records obtained for each experimental concentration and each inhibitor. **(F,G)** Inhibition by T16A_inh_-A01 (10 μM, *n* = 4; 30 μM, *n* = 8) and NFA (10 μM, *n* = 4; 30 μM, *n* = 12; 100 μM, *n* = 8) of RVD following 100 mOsm.l^-1^ hypotonic challenge based on calcein relative fluorescence.

Finally, we tested two inhibitors, T16A_inh_-A01 and NFA, that are known to target ANO1/CaCC conductance. Interestingly, T16A_inh_-A01 induced a marked decrease in hypotonically activated Cl^-^ currents at a concentration of 3 μM (23 ± 5% inhibition, *n* = 4, **Figures 5A,B**). NFA also significantly inhibited the hypotonically activated Cl^-^ current at a concentration of 10 μM (23 ± 4% inhibition, **Figure 5A**), and a maximal inhibitory effect was obtained at 100 μM (**Figure 5C**). In whole-cell experiments, the IC_50_ values for T16A_inh_-A01 and NFA were calculated as 5.5 ± 1.4 μM (*n* = 4, **Figure 5D**) and 55.2 ± 2.2 μM (*n* = 5, **Figure 5E**), respectively.

Using fluorescent RVD quantification, we observed that T16A_inh_-A01 inhibited the RVD process at 10 μM (40 ± 10%, *n* = 7) and that it had a maximal effect at 30 μM (**Figure 5F**). NFA showed a slight effect at 10 μM (20 ± 7%, *n* = 6) and maximally inhibited the RVD process at 30 μM (**Figure 5G**).

## Discussion

For decades, chloride channels have been studied using non-specific inhibitors; only since 2000 some “specific” inhibitors have been identified using high-throughput screening assays (Jayaraman et al., 2000; Galietta et al., 2001). Over the same time period, the molecular identification of two families of chloride channels has emerged. Bestrophin and anoctamin have been shown to form calcium-activated chloride channels (CaCC), whereas LRRC8 family members have been demonstrated to be essential components of the VRAC. However, the roles and functions of these channels remain unclear, and most studies to date have been based on the use of so-called “specific” inhibitors.

In this study, we measured the inhibitory effect of 3 CFTR inhibitors and 2 CaCC inhibitors on VRAC conductance both by patch-clamp recording and by measurement of the RVD in cells lacking CFTR and CaCC. **Table 1** summarizes the IC_50_, the efficacy at 10 μM in whole-cell and RVD experiments and the corresponding calculated Hill coefficient for all inhibitors. Indeed, the results of qPCR experiments showed that CFTR, TMEM16A and TMEM16B are not expressed in HEK-293 cells. These cells mainly expressed LRRC8A and LRRC8D, which form VRAC, and three members of TMEM16: TMEM16E (ANO5), TMEM16F (ANO6) and TMEM16K (ANO10). Little is known about TMEM16E and TMEM16K (Pedemonte and Galietta, 2014). TMEM16E does not form an ion channel, whereas TMEM16K has been proposed to be a chloride channel, although it seems to be localized in an intracellular compartment. TMEM16F has been reported to act both as a scramblase and as a chloride channel that is activated by micromolar concentrations of calcium (Shimizu et al., 2013). Although patch-clamp recording confirmed the absence of CFTR and CaCC conductance in our experiments, we cannot exclude the possibility that TMEM16F functions as a CaCC in HEK-293 RVD measurements. Indeed, Juul et al. (2014) first showed that TMEM16F differs from VRAC but supports volume regulation in the presence of calcium. A recent study confirmed the role of TMEM16F in volume regulation and proposed that it could be an osmosensor that is in close proximity to VRAC (Sirianant et al., 2016a).

**Table 1:** Effectiveness parameters of the six chloride channel inhibitors obtained from whole-cell experimental recordings and RVD experiments.

	Chloride conductance				RVD	
	IC_50_ (μM)	Hill coefficient	*n*	Inhibition at 10 μM (%)	Inhibition at 10 μM (%)	
DCPIB	4.8 ± 1.2	2.9 ± 1.1	*n* = 5 (*R*^2^> 0.9)	82.7 ± 3.2	35.8 ± 3.1	*n* = 8
CFTR_inh_-172	No effect	–	*n* = 5	No effect	No effect	*n* = 10
GlyH-101	9.5 ± 1.1	0.9 ± 0.4	*n* = 5 (*R*^2^ > 0.9)	46.6 ± 8.8	NA	*n* = 8
PPQ-102	19.6 ± 1.5	2.2 ± 1.2	*n* = 5 (*R*^2^> 0.7)	18.3 ± 11.3	29.8 ± 5.9	*n* = 12
NFA	55.2 ± 2.2	0.8 ± 0.4	*n* = 5 (*R*^2^ > 0.9)	22.5 ± 4.0	19.9 ± 7.2	*n* = 6
T16A_inh_-A01	5.5 ± 1.4	1.5 ± 0.6	*n* = 4 (*R*^2^ > 0.9)	58.4 ± 2.0	39.8 ± 9.6	*n* = 7

*The table shows the IC_*50*_, the Hill coefficient, and the percent inhibition of VRAC/LRRC8 conductance measured for each inhibitor at a fixed concentration of 10 μM. For comparison, the percent inhibition measured in calcein-based RVD experiments in which the inhibitors were used at a fixed concentration of 10 μM is also given.*

### DCPIB: Specificity and Limits

Synthesized in 2001, DCPIB rapidly became a gold standard for VRAC due to its apparent specificity. Indeed, Decher et al. (2001) reported that it blocks more than 80% of I_Cl,swell_ at 10 μM without any effect on other chloride channels (CFTR, CaCC, ClC). However, few reports have sought to identify other targets of DCPIB. It has been shown to activate TREK channels in astrocytes, to block some K_ir_ channels and to inhibit gastric H/K-ATPase (Minieri et al., 2013; Fujii et al., 2015; Deng et al., 2016). Interestingly, DCPIB was recently identified as a potent inhibitor of glutamate transport pathways in glial cells and of connexin hemichannels (Cx43) (Ye et al., 2009; Bowens et al., 2013). Retrospectively, the latter result is not surprising because connexins exhibit close structural homology with VRAC/LRRC8 members (Abascal and Zardoya, 2012).

In the present work, we measured an IC_50_ value for DCPIB that is close to the value reported by Decher et al. (2001) and confirmed that DCPIB is the most effective VRAC-blocking drug that has so far been tested. Despite its IC_50_ of 4.8 μM, this compound should be used carefully in light of its putative efficiency on connexin and pannexin. Specifically, the connexin hemichannel and pannexin are involved in the transport of ATP and organic osmolytes, and the role of VRAC in this process remains an ongoing and controversial question (Sabirov and Okada, 2005).

### CFTR Inhibitors

Cystic fibrosis transmembrane conductance regulator has been widely studied in the context of cystic fibrosis. This cAMP-dependent chloride channel is mainly expressed in epithelial tissues, and it is involved in ion and fluid secretion in several organs such as the lung, the intestinal tract, the pancreas and the kidney. In this study, we evaluated the effects of three known CFTR inhibitors on VRAC conductance in cells lacking CFTR. CFTR_inh_-172, which was discovered in 2002, is the most used CFTR inhibitor; it has an IC_50_ of 300 nM and is commonly used at 10 μM. Here, we observed no significant effect of CFTR_inh_-172 on VRAC current or RVD at concentrations of up to 20 μM. These results are consistent with results reported previously (Ma et al., 2002), even considering that we reported an inhibition of VRAC current in mouse kidney and hamster PS120 cells at CFTR_inh_-172 concentrations higher than 5 μM (Melis et al., 2014). This discrepancy might be due to species differences in channel sensitivity. Such a difference for a single concentration of inhibitor has already been described for CFTR orthologs from four different species (Stahl et al., 2012). Second, we confirmed the results obtained for GlyH-101 in our previous study, in which we showed that GlyH-101 is a potent inhibitor of I_Cl,swell_ (Melis et al., 2014). Discovered in 2004, GlyH-101 exhibits an IC_50_ for CFTR of approximately 5 μM and is commonly used at concentrations ranging from 10 to 100 μM (Muanprasat et al., 2004). We measured an IC_50_ value for GlyH-101 of 9.5 μM and showed that I_Cl,swell_ was inhibited by more than 80% in the presence of 20 μM GlyH-101. Finally, the last tested CFTR inhibitor was PPQ-102, which was discovered in 2009 (Tradtrantip et al., 2009). Surprisingly, this inhibitor is rarely used, even though it has an IC_50_ value below 100 nM. We showed that this drug also targets VRAC conductance with an IC_50_ of 20 μM and that it inhibited I_Cl,swell_ by more than 70% at a concentration of 30 μM but was ineffective at 1 μM. This drug impaired RVD in a dose-dependent manner, but only at concentrations higher than 10 μM. Nevertheless, it might be possible to use PPQ-102 at low concentrations (<1 μM), a relatively low concentration that is known to induce a marked inhibition of CFTR and to exhibit negligible effects on VRAC.

### CaCC Inhibitors

Calcium-activated Cl^-^ conductance has been described in a wide variety of tissues, including intestinal, airway, pancreas and renal epithelial cells, as well as in smooth muscle cells and sensory neurons. This chloride channel is activated by an intracellular increase in free calcium, and it is generally thought that the flux of Cl^-^ ions through the CaCC channel is driven by ANO1 or ANO2. Before the molecular identification of the ANO family as a main constituent of CaCC, NFA was known to inhibit both CaCC and VRAC, and it is often used at a concentration of 100 μM. We report here in the HEK-293 cell line devoid of ANO1 or 2 expression and CaCC conductance that NFA inhibits the VRAC in a dose-dependent manner with an IC_50_ of 55 μM, as expected.

In addition to NFA and its dual action on CaCC and VRAC, the more specific inhibitor T16A_inh_-A01 has become the gold standard for targeting CaCC/ANO-1 conductance. T16A_inh_-A01 was first synthesized in 2011, and the seminal study of Namkung et al. (2013) reported that it has an IC_50_ of 1 μM for CaCC. Here, we showed for the first time that T16A_inh_-A01 also inhibits VRAC conductance and the RVD process. Using patch-clamp experiments, we calculated an IC_50_ value for T16A_inh_-A01 of 5.5 μM, a value very close to the value obtained previously for CaCC/ANO1 inhibition. This result excludes the use of this inhibitor to discriminate the contribution of CaCC from that of VRAC in cells that express both ANO1-2 and LRRC8 proteins. Moreover, the functional interrelationship between CaCC/ANO1 and the ubiquitous VRAC/LRRC8, as shown in a series of studies from the Kunzelmann laboratory (Benedetto et al., 2016; Sirianant et al., 2016a,b), suggests that this inhibitor should be used with great caution.

Interestingly, in our experiments with HEK-293 cells, CaCC inhibitors were more effective in blocking RVD than other inhibitors, indicating that VRAC conductance is probably not the only mechanism involved in RVD even if full blockade of I_Cl,swell_ by 20 μM DCPIB or knockdown of LRRC8A expression completely impaired volume regulation. It is possible that TMEM16F or TMEM16K is also involved and that these channels participate in I_Cl,swell_ development; if this is the case, CaCC inhibitors might work better due to their complementary actions. Alternatively, these drugs might act through another related mechanism; T16A_inh_-A01 has been reported to have a wide range of non-specific effects (Boedtkjer et al., 2015).

Despite the progress that has been made in the molecular identification of chloride channel families, most inhibitors of these channels, including fenamate, niflumate, T16A_inh_-A01, CaCC_inh_-A01 and NS3728, are still non-specific and indiscriminately target both VRAC/LRRC8 and CaCC/ANO1-2. Recently, Seo et al. (2016) identified a new inhibitor, Ani9 that seems to mainly inhibit TMEM16A current with negligible effects on CFTR, VRAC and TMEM16B.

**FIGURE 6:**
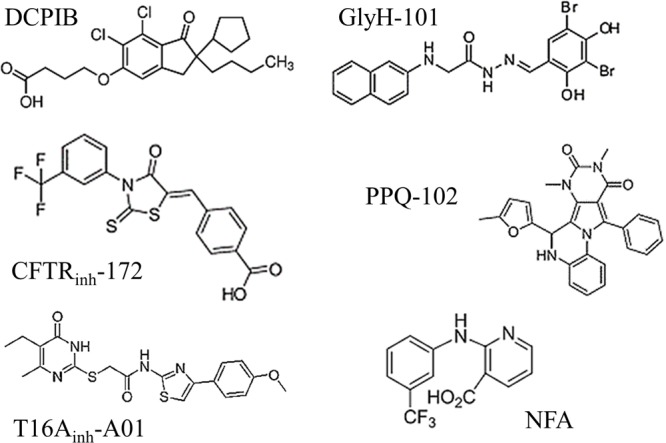
Molecular structures of the six chloride channel inhibitors used in this study. Note that none of these drugs share any evident structural homologies.

A comparison of the molecular structures of the six tested inhibitors (**Figure 6**) does not reveal any evident structural homologies among them; however, Hill slope analysis reveals different profiles in their inhibitory mechanisms (**Table 1**). The Hill coefficients of GlyH-101 and NFA are close to one, suggesting that those compounds display no cooperativity. Conversely, DCPIB, PPQ-102 and T16A_inh_-A01 have Hill coefficients greater than 1 (2.9 ± 1.1, 2.2 ± 1.2, and 1.5 ± 0.6, respectively), indicating that VRAC might possess multiple binding sites for these drugs, resulting in cooperativity.

Finally, of the 6 chloride channel inhibitors tested, DCPIB remains one of the most “potent and specific” drugs available to inhibit VRAC/LRRC8 conductance despite the high working concentration needed (>10 μM). Unfortunately, the other inhibitors that have been reported to target CFTR (GlyH-101, PPQ-102) or CaCC/ANO1-2 (T16A_inh_-A01, NFA) also inhibited VRAC/LRRC8 conductance over almost the same concentration ranges. CFTR_inh_-172 appears to be the most specific inhibitor for CFTR, showing only minor action on human VRAC/LRRC8 even at high concentrations.

The lack of specificity of the chloride channel inhibitors we have tested emphasizes the need to be very cautious regarding their use in research. In conclusion, further investigation should be conducted on the specificity of chloride channel inhibitors so as not to falsely attribute effects to one channel versus another.

## Author Contributions

JF, MT, MC, IR, and CD performed the cellular experiments and the quantitative PCR experiments. JF and CD performed the patch-clamp experiments. IR, CD, and VC wrote the paper with input and discussion from all of the co-authors.

## Supplementary Material

The Supplementary Material for this article can be found online at: http://journal.frontiersin.org/article/10.3389/fphar.2017.00328/full#supplementary-material

FIGURE S1 Absence of CFTR and CaCC conductance in wild-type HEK-293 cells. **(A)** Representative whole-cell chloride currents recorded before (basal) and after addition of 10 μM forskolin and 100 μM IBMX (*n* = 5). **(B)** Representative whole-cell chloride currents recorded before (basal) and after addition of 1 μM ionomycin (*n* = 5). Ionomycin, a specific Ca^2+^ ionophore, failed to trigger any calcium-activated chloride current **(B)**, whereas it induced an increase in intracellular calcium. **(C)** Variations in intracellular calcium concentration induced by exposure to ionomycin (plate reader measurements). Cells were incubated for 45 min with a fluorescent Ca^2^ -sensitive probe (Quest Fluo-8), maintained in HBSS-like solution containing 1 mM CaCl_2_ and exposed to ionomycin (1 μM). At the end of the experiment, *F*_min_ fluorescence values were obtained by exposing the cells to an HBSS-like calcium-free solution containing 12 mM EGTA. The values shown are the mean ±SEM of 12 independent experiments.

FIGURE S2 Regulatory volume decrease as a function of hypotonic challenge intensity and polylysine coating of the plastic support. Experiments were performed using calcein relative fluorescence quenching induced by changes in cellular volume. The fluorescence emitted at 520 nm was measured every 90 s over a 60-min period. **(A)** After a 10-min period for fluorescence stabilization, the bath solution was replaced by an iso-osmotic solution (300 mOsm.l^-1^) or by various hypo-osmotic solutions (200, 150, or 100 mOsm.l^-1^). The reported values are the mean ±SEM of 6 individual experiments for each experimental condition. **(B)** Quantification of regulatory volume decrease following a hypotonic challenge (100 mOsm.l^-1^) performed on cells cultured on plastic supports that were coated or not with polylysine. The values shown are the mean ±SEM of 6 individual experiments.

FIGURE S3 Absence of CFTR_inh_-172-mediated inhibitory effect on I_Cl,swell_. **(A)** Mean current/voltage relationships measured 10 ms after the onset pulse corresponding to experiments performed in **Figure 4** in the absence or presence CFTR_inh_-172 (20 μM, *n* = 5 of individual records). **(B)** Dose-response inhibition curve calculated from whole-cell current recordings of cells exposed to 1, 10, and 20 μM CFTR_inh_-172. The percent inhibition induced by CFTR_inh_-172 at -100 mV was calculated for each concentration (*n* = 5 for each experimental concentration).

FIGURE S4 Basal fluorescence of GlyH-101-impeded regulatory volume decrease measurement. The calcein relative fluorescence method is impeded by the basal auto-fluorescence of GlyH-101 (10 μM, *n* = 8). Experiments were performed without hypotonic challenge; the same iso-osmotic solution (300 mOsm.l^-1^) was maintained during all of the experiments.

## Conflict of Interest Statement

The authors declare that the research was conducted in the absence of any commercial or financial relationships that could be construed as a potential conflict of interest.

## Footnotes

1 http://www.graphpad.com

2 http://www.R-project.org/

## Abbreviations

CaCC: calcium-activated Cl^-^ conductance
CFTR: cystic fibrosis transmembrane conductance regulator
LRRC8: leucine-rich repeat containing 8
RVD: regulatory volume decrease
TMEM16: transmembrane protein 16
VRAC: volume-regulated anion channel.
