# Supplementary figures and images for: Comparative Effects of Chloride Channel Inhibitors on LRRC8/VRAC-Mediated Chloride Conductance
Source: Front Pharmacol. 2017 May 31;8:328. doi: 10.3389/fphar.2017.00328 (PMC5449500; doi:10.3389/fphar.2017.00328)

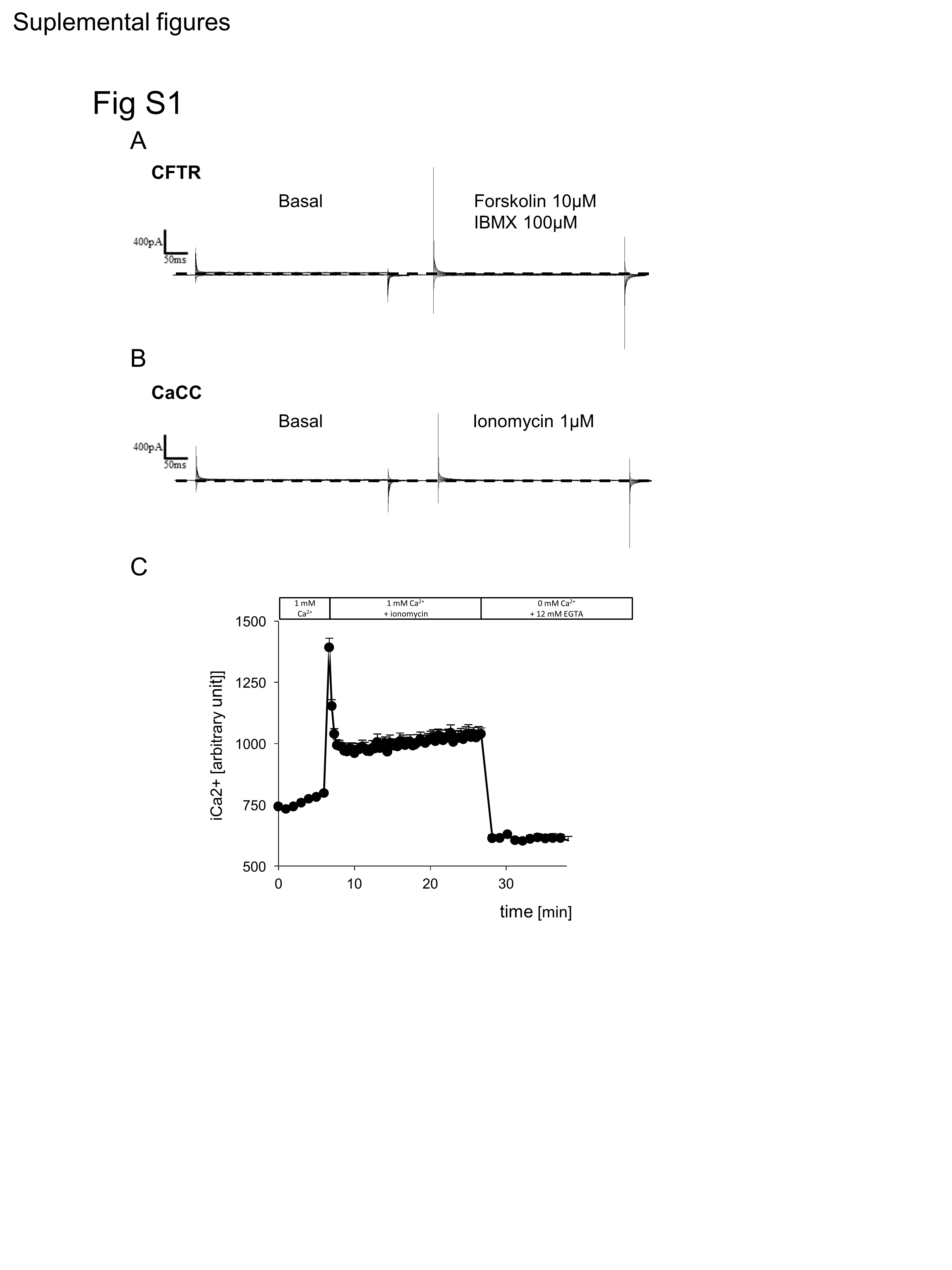

Supplement: FIGURE S1 — Absence of CFTR and CaCC conductance in wild-type HEK-293 cells. (A) Representative whole-cell chloride currents recorded before (basal) and after addition of 10 μM forskolin and 100 μM IBMX (n = 5). (B) Representative whole-cell chloride currents recorded before (basal) and after addition of 1 μM ionomycin (n = 5). Ionomycin, a specific Ca2+ ionophore, failed to trigger any calcium-activated chloride current (B), whereas it induced an increase in intracellular calcium. (C) Variations in intracellular calcium concentration induced by exposure to ionomycin (plate reader measurements). Cells were incubated for 45 min with a fluorescent Ca2 -sensitive probe (Quest Fluo-8), maintained in HBSS-like solution containing 1 mM CaCl2 and exposed to ionomycin (1 μM). At the end of the experiment, Fmin fluorescence values were obtained by exposing the cells to an HBSS-like calcium-free solution containing 12 mM EGTA. The values shown are the mean ±SEM of 12 independent experiments. [file Image_1.JPEG]

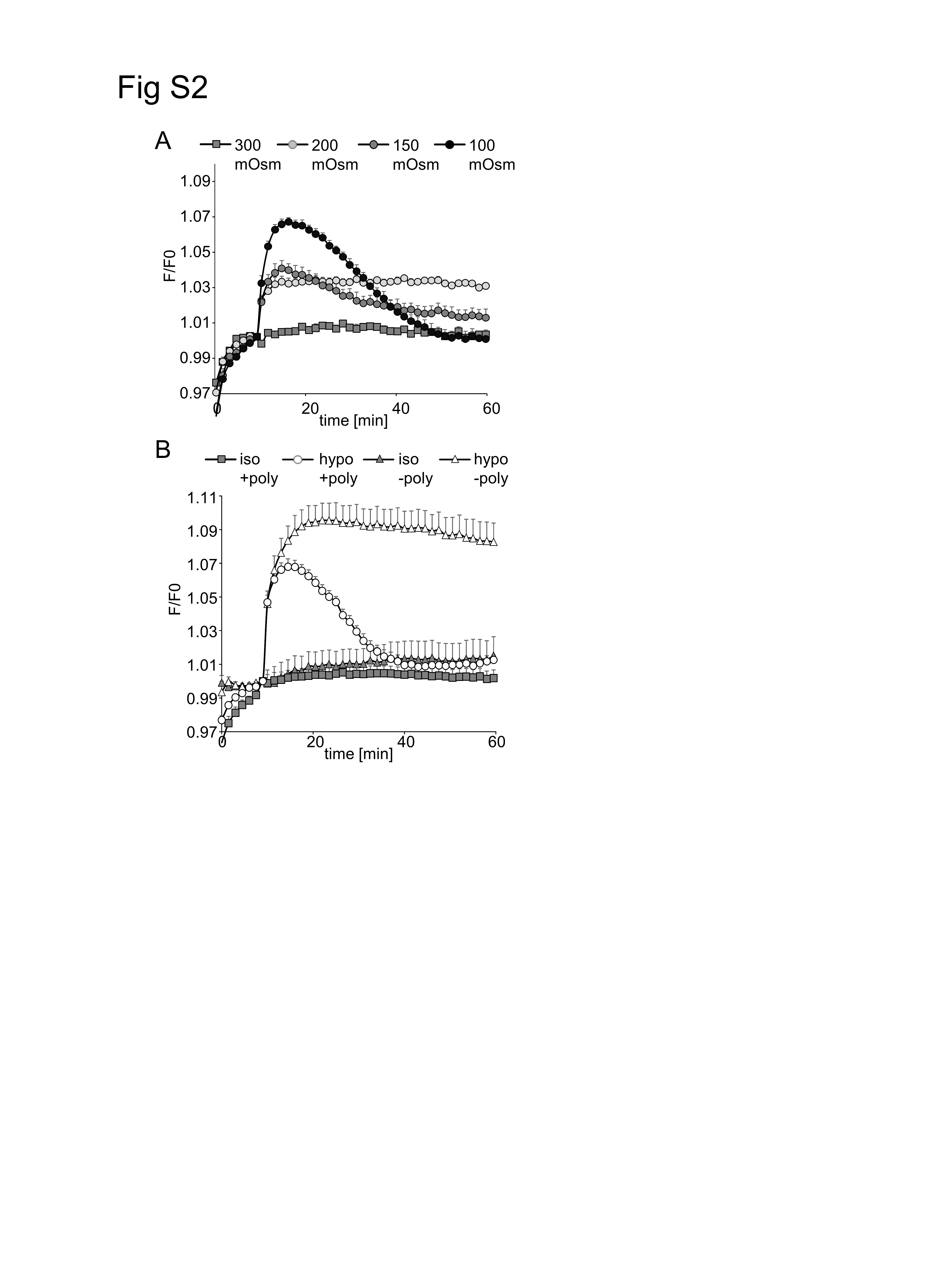

Supplement: FIGURE S2 — Regulatory volume decrease as a function of hypotonic challenge intensity and polylysine coating of the plastic support. Experiments were performed using calcein relative fluorescence quenching induced by changes in cellular volume. The fluorescence emitted at 520 nm was measured every 90 s over a 60-min period. (A) After a 10-min period for fluorescence stabilization, the bath solution was replaced by an iso-osmotic solution (300 mOsm.l-1) or by various hypo-osmotic solutions (200, 150, or 100 mOsm.l-1). The reported values are the mean ±SEM of 6 individual experiments for each experimental condition. (B) Quantification of regulatory volume decrease following a hypotonic challenge (100 mOsm.l-1) performed on cells cultured on plastic supports that were coated or not with polylysine. The values shown are the mean ±SEM of 6 individual experiments. [file Image_2.JPEG]

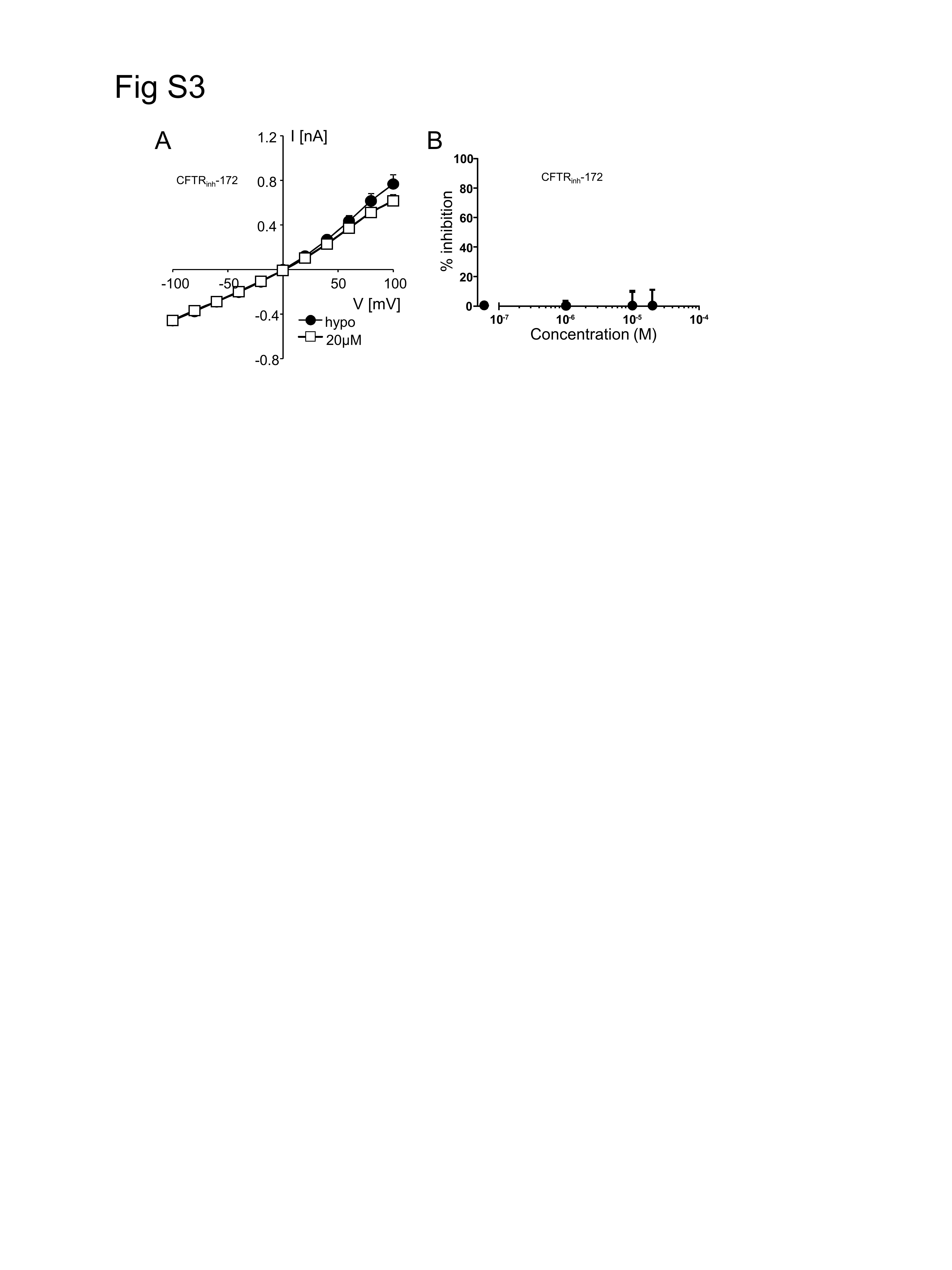

Supplement: FIGURE S3 — Absence of CFTRinh-172-mediated inhibitory effect on ICl,swell. (A) Mean current/voltage relationships measured 10 ms after the onset pulse corresponding to experiments performed in Figure 4 in the absence or presence CFTRinh-172 (20 μM, n = 5 of individual records). (B) Dose-response inhibition curve calculated from whole-cell current recordings of cells exposed to 1, 10, and 20 μM CFTRinh-172. The percent inhibition induced by CFTRinh-172 at -100 mV was calculated for each concentration (n = 5 for each experimental concentration). [file Image_3.JPEG]

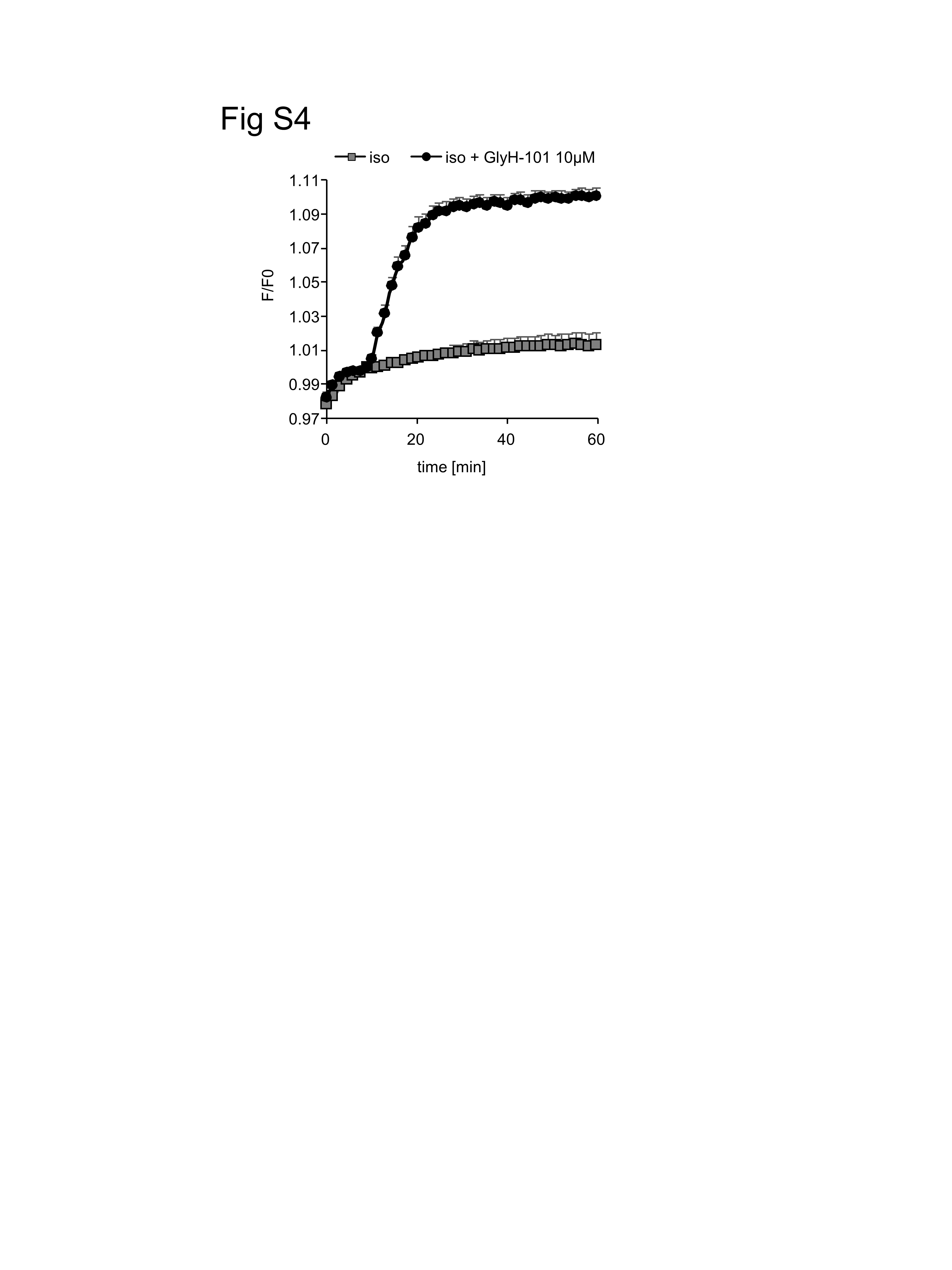

Supplement: FIGURE S4 — Basal fluorescence of GlyH-101-impeded regulatory volume decrease measurement. The calcein relative fluorescence method is impeded by the basal auto-fluorescence of GlyH-101 (10 μM, n = 8). Experiments were performed without hypotonic challenge; the same iso-osmotic solution (300 mOsm.l-1) was maintained during all of the experiments. [file Image_4.JPEG]
